# Short-term clinical outcomes of 42 cases of arthroscopic meniscectomy for discoid lateral meniscus tears

**DOI:** 10.3892/etm.2012.686

**Published:** 2012-08-28

**Authors:** HONG CAO, YING ZHANG, WEI QIAN, XIN-HUA CHENG, YONG KE, XIAO-PENG GUO

**Affiliations:** 1 Department of Orthopedic Surgery and; 2 Reproductive Medicine Center, Renmin Hospital, Hubei University of Medicine, Shiyan, Hubei 442000, P.R. China

**Keywords:** discoid lateral meniscus, arthroscopic, meniscectomy

## Abstract

Discoid lateral meniscus of the knee causes a high morbidity in China. Since the traditional treatment to open the capsule and resect the meniscus often results in arthritis, it is now believed that a discoid lateral meniscus should be treated with arthroscopy to preserve part of the meniscus. The current study aimed to investigate the short-term clinical outcomes of arthroscopic meniscectomy for the treatment of discoid lateral meniscus tears. In the present study, we diagnosed and treated 42 patients (47 knees) with discoid lateral meniscus tears using arthroscopy between February, 2007 and December, 2010. Thirty-seven knees received partial resection of the discoid meniscus, 8 received hypo-complete resection and 2 received complete resection. Thirty-nine of the patients were followed up for a mean of 21 months (ranging from 9 to 53 months). The Lysholm scoring system was used to assess the knee function prior to surgery and during the follow-up. The results were analyzed using a Student’s t-test with SPSS 12.0. Our study showed that patients with treated knees returned to normal activities within 4–6 weeks, and knee functions were more improved at 9 months after operation than 3 months, as measured by the Lysholm score (P<0.05). Arthroscopic meniscectomy is an effective treatment for discoid menisci resulting in minimal invasion, quick recovery and early functional exercise. The use of arthroscopy during surgery aids to preserve the meniscus and to reduce stress, therefore, having a beneficial effect on short-term clinical outcomes.

## Introduction

A discoid meniscus is an anatomical congenital anomaly which was considered to be a vestige of viviparous cartilage development of the knee (1,2). Diagnosis of the discoid meniscus has been improved by the MRI technique, and the incidence of discoid lateral meniscus of the knee in the Chinese population is 16–46% (3). Although it may be left untreated, tearing of the discoid lateral meniscus may cause pain and immobility of the knee joint, and therefore requires surgery. The traditional treatment is to open the capsule and resect the meniscus, but this often leads to the development of arthritis (4). Since the use of arthroscopy has been suggested to preserve part of the meniscus (5,6), we investigated the short-term clinical outcomes of 43 cases of arthoscopic meniscectomy for discoid lateral meniscus tears.

## Patients and methods

### Patients

The study was conducted in the Renmin Hospital, Hubei University of Medicine, China from February, 2007 to December, 2010. Forty-two patients (47 knees) with injured discoid lateral meniscus were treated using arthroscopy, including 10 men and 32 women, aged from 14 to 62 years (mean, 31.46). The type of discoid lateral meniscus in these cases was evaluated by the O’Connor classification (7) (Table I) and there was no Wrisberg-type by Watanabe classification (8). This study was approved by the ethics committee of Hubei University of Medicine Hubei, China) and written informed consent was obtained from all subjects.

**Figure 1:**
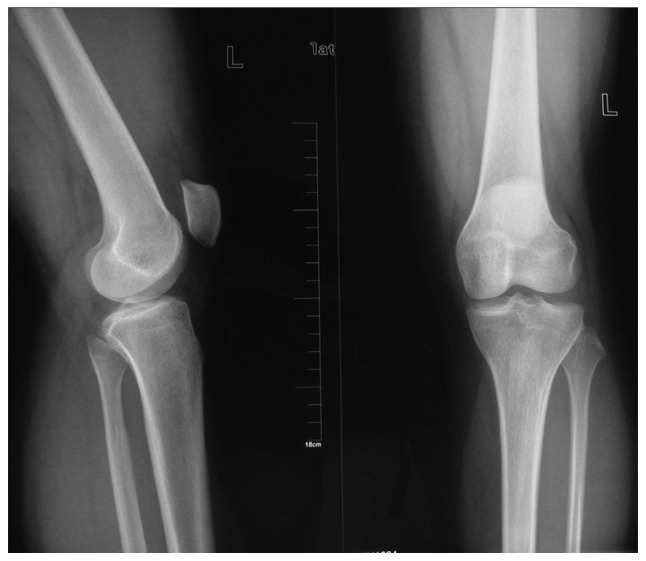
X-ray imaging prior to surgery shows a widened lateral joint space.

**Figure 2:**
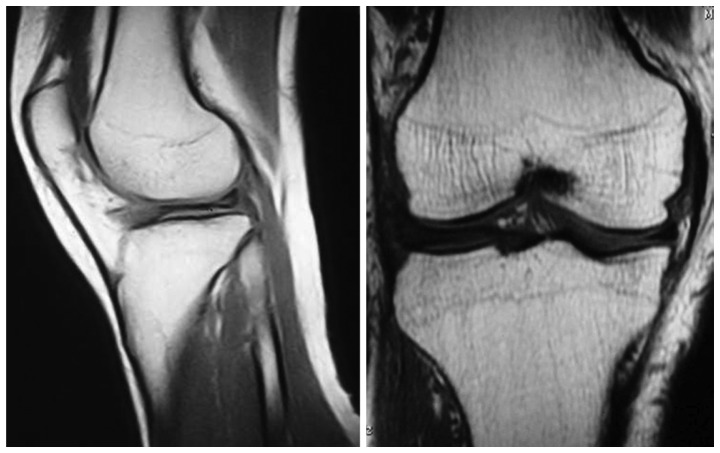
MRI shows injured discoid lateral meniscus.

The preoperative examinations included physical examination, X-ray imaging and MRI of the injured knee. Physical examination revealed atrophy of the quadriceps femoris muscle, lateral tibiofemoral joint line tenderness, restriction of mobility and positive McMurray sign. Certain patients had ‘clicking’ of joints. Radiography revealed a widened lateral joint space (Fig. 1) in 13 knees. The MRI results were all in accordance with those of the arthroscopic examination (Fig. 2).

### Surgical techniques

**Figure 3:**
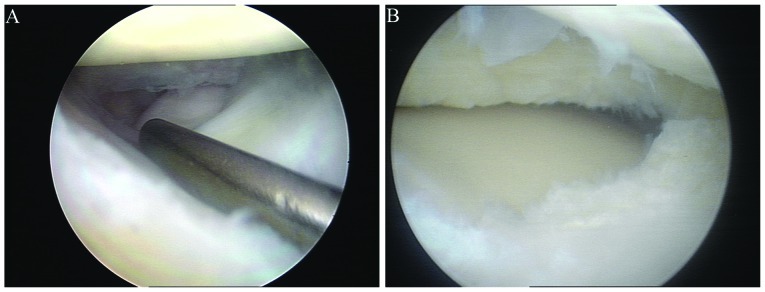
(A) Discoid lateral meniscus with longitudinal tear, (B) discoid lateral meniscus following partial resection.

**Figure 4:**
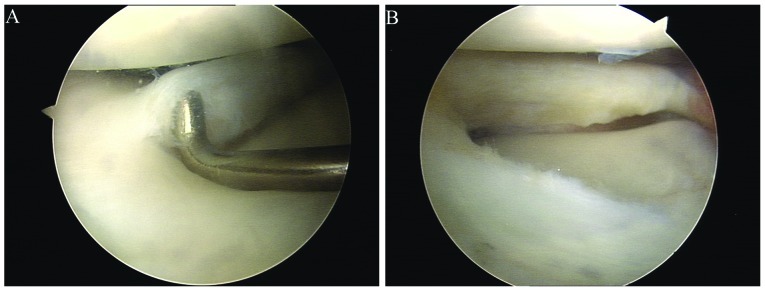
(A) Discoid lateral meniscus with simple horizontal tear, (B) discoid lateral meniscus following surgery.

Patients were arthroscoped (Stryker, Kalamazoo, MI, USA) in the supine position under continuous peridural anesthesia or combined spinal epidural anesthesia with a calibrated pneumatic tourniquet (the tourniquet time was <90 min). Arthroscopic examination was performed to observe the intra-articular structures in the following order: suprapatellar pouch, patellofemoral joint, medial gutter, medial compartment, intercondylar notch, lateral compartment and lateral gutter. The meniscus was probed carefully to identify individual structures, type of the discoid lateral meniscus, stability of the peripheral rim, position and extent of the meniscus tear, as well as other accompanying lesions (Figs. 3A and 4A).

The meniscal tear was carefully resected using standard techniques and the meniscal rim was preserved. The methods in common use are partial resection (shaping of the discoid meniscus), hypo-complete resection and complete resection. For partial meniscectomy, the small inferior leaf of the horizontal cleavage tear was partially resected, whereas the main body was preserved 6–8 mm in width during surgery (9) and the anatomical shape was maintained with arthroscopy. Following the meniscectomy, the resected edge was smoothened, the meniscus was reshaped (from discoid to crescent), the peripheral rim was then thickened and the free edge of the meniscus was thinned to form a slope (Figs. 3B and 4B). Following surgery, the joint was lavaged thoroughly to remove all the debris, the arthroscopic portals were sutured and the knee was compressed with a bandage.

### Postoperative rehabilitation

The rehabilitation training programs started soon after surgery with all the patients instructed to perform isometric quadricep exercises. The muscular training in the first week following surgery was focused on the quadriceps femoris muscle, including straight leg raises and Actimove GenuFlex movements, but no weight loading. Active flexion and extension exercises of the knee joint were performed in the second week, and patients could walk with walking sticks. Dermal sutures were removed 14 days after surgery, and four weeks after surgery, patients went back to normal life and continued the above training.

### Follow-up

Thirty-nine of the patients (43 knees) were followed up for a mean of 21 months (ranging from 9 to 53 months). The Lysholm scoring system (10,11) was used to assess the function of the knee prior to surgery and during the follow-up, and the results were compared using a Student’s t-test with SPSS 12.0.

## Results

Among the 47 knees, 37 received partial resection, 8 received hypo-complete resection and 2 had complete resection (Table II).

One patient had pain and swelling of the knee joint postoperation, but the symptom disappeared 4 months after surgery. Another patient suffered with hemarthrosis, and the symptom disappeared following arthrocentesis. All the patients were instructed to perform the rehabilitation training and returned to normal activities within 4–6 weeks.

Knee function significantly improved postoperation, and the clinical outcome was improved at 9 months compared to the function after 3 months, as measured by Lysholm score (P<0.05), showing the curative effect of meniscectomy (Table III).

## Discussion

Discoid lateral meniscus of the knee is common in Asian populations (12,13). Unlike normal menisci, discoid menisci cannot control the coordination of the tibiofemoral joint, absorb shock, or reduce the mechanical pressure on articular cartilage, thus they quickly become worn and are torn easily, particularly when injured (14). Atay *et al* (15) revealed that the ultrastructure of discoid lateral menisci significantly differs from that of normal menisci. The collagen fibrils in discoid menisci are decreased in number and misaligned, both of which contribute to an increased incidence of tears. Therefore we suggested discoid menisci be treated by arthroscopy early, even when asymptomatic.

MRI accurately displays a discoid meniscus and the type, extent and position of the tear (16). In the present study, the use of radiography in addition to MRI was useful, since it identifies and tracks changes in the bone before and after surgery, including osteoarthritis, rheumatoid arthritis, fracture and bone tumor. We identified a widened lateral joint space in 13 knees by radiography.

The traditional treatment for a discoid lateral meniscus tear is to open the capsule and resect the meniscus, but this often leads to arthritis development, particularly in children who receive a total meniscectomy (17). With the recent advance in arthoscopic surgical techniques and results from research on healing function of meniscus arthroscopy, meniscal repair has now become the technique of first choice to preserve menisci (18). The aim of the surgery is to remove the central and torn parts of the discoid meniscus and to preserve a stable peripheral rim as much as possible. Since the thick discoid lateral meniscus is located within the space between knee joints this affects the performance of the surgery, and a large quantity of meniscal tissues has to be removed. It is much more difficult to perform the shaping of the discoid meniscus (partial resection) for a discoid lateral meniscus than for a normal one. In our experience, bending the knee during the surgery and lowering the lower leg along the side of the operation table to open up the joint space via gravity, as well as using a suitable meniscus knife and meniscus scissors, was helpful. When the discoid meniscus is reshaped, the femoral surface should be resected more to form a slope adapting to the shape of the femoral condyles. In our study, among the 47 knees, 37 received partial resection (78.72%), 8 received hypo-complete resection (17.02%) and 2 received complete resection (4.26%). Hayashi *et al* (9) suggested that the rim should be retained to 6–8 mm in width, but the excessive thickness of a complete-type discoid meniscus should be reduced substantially to avoid new tears. In partial meniscectomies, a rim of 8 mm was originally left for complete-type lesions and 10 mm for incomplete-type lesions (the average width of normal menisci is 12–13 mm). We followed this standard in our study.

In conclusion, arthroscopic meniscectomy is an effective treatment for discoid menisci resulting in maximal meniscus preservation, minimal invasion, quick recovery and early functional exercise.

## Figures and Tables

**Table I:** Tear patterns according to types of discoid lateral meniscus.

	Type of discoid lateral meniscus		
Tear pattern	Complete	Incomplete	Total
Simple horizontal	7	0	7
Complicated horizontal	8	4	12
Longitudinal	7	6	13
Radial	0	8	8
Degenerative	0	4	4
Complex	0	3	3
Total	22	25	47

**Table II:** Surgical methods according to types of tear patterns.

	Type of surgical method			
Tear pattern	Partial resection	Hypo-complete resection	Complete resection	Total
Simple horizontal	6	0	1	7
Complicated horizontal	9	3	0	12
Longitudinal	10	2	1	13
Radial	6	2	0	8
Degenerative	4	0	0	4
Complex	2	1	0	3
Total	37	8	2	47

**Table III:** The Lysholm score measured preoperatively and postoperatively.

Time	Lysholm score
Preoperative	66.83±8.26
3 months after operation	91.48±3.01^a^
9 months after operation	95.28±2.01^b^

a Knee functions were significantly improved 3 months after surgery as measured by Lysholm score compared to preoperative functions (P<0.05).

b The clinical outcomes were improved after 9 months compared to 3 months (P<0.05).
